# Discovery of DNA Viruses in Wild-Caught Mosquitoes Using Small RNA High throughput Sequencing

**DOI:** 10.1371/journal.pone.0024758

**Published:** 2011-09-20

**Authors:** Maijuan Ma, Yong Huang, Zhengda Gong, Lu Zhuang, Cun Li, Hong Yang, Yigang Tong, Wei Liu, Wuchun Cao

**Affiliations:** 1 State Key Laboratory of Pathogen and Biosecurity, Beijing Institute of Microbiology and Epidemiology, Beijing, China; 2 Yunnan Institute of Endemic Disease Control and Prevention, Dali, Yunnan, China; New Mexico State University, United States of America

## Abstract

**Background:**

Mosquito-borne infectious diseases pose a severe threat to public health in many areas of the world. Current methods for pathogen detection and surveillance are usually dependent on prior knowledge of the etiologic agents involved. Hence, efficient approaches are required for screening wild mosquito populations to detect known and unknown pathogens.

**Methodology/principal findings:**

In this study, we explored the use of Next Generation Sequencing to identify viral agents in wild-caught mosquitoes. We extracted total RNA from different mosquito species from South China. Small 18–30 bp length RNA molecules were purified, reverse-transcribed into cDNA and sequenced using Illumina GAIIx instrumentation. Bioinformatic analyses to identify putative viral agents were conducted and the results confirmed by PCR. We identified a non-enveloped single-stranded DNA densovirus in the wild-caught *Culex pipiens molestus* mosquitoes. The majority of the viral transcripts (.>80% of the region) were covered by the small viral RNAs, with a few peaks of very high coverage obtained. The +/− strand sequence ratio of the small RNAs was approximately 7∶1, indicating that the molecules were mainly derived from the viral RNA transcripts. The small viral RNAs overlapped, enabling contig assembly of the viral genome sequence. We identified some small RNAs in the reverse repeat regions of the viral 5′- and 3′ -untranslated regions where no transcripts were expected.

**Conclusions/significance:**

Our results demonstrate for the first time that high throughput sequencing of small RNA is feasible for identifying viral agents in wild-caught mosquitoes. Our results show that it is possible to detect DNA viruses by sequencing the small RNAs obtained from insects, although the underlying mechanism of small viral RNA biogenesis is unclear. Our data and those of other researchers show that high throughput small RNA sequencing can be used for pathogen surveillance in wild mosquito vectors.

## Introduction

Emerging infectious diseases (EIDs) have exerted a significant burden on public health and global economies [1], [2]. During the past decade, novel viruses, particularly those causing severe acute respiratory syndrome (SARS) and avian influenza A H5N1, have attracted international concern. These diseases represent only part of a rich tapestry of pathogens that have emerged to pose public health threats in recent years. Clearly, there is a pressing need for rapid and accurate identification of viral etiological agents. The development of Next Generation Sequencing (high throughput sequencing) technology provides a possible solution to this problem; indeed several recent studies have used these techniques to identify novel viral agents [3], [4], [5], [6], [7]. Palacios *et al.* identified a novel and deadly arenavirus by employing 454-pyrosequencing technology, the results of which were later confirmed by PCR [4]. Recent studies, have identified a novel strain of Ebola virus which caused a hemorrhagic fever epidemic in Uganda [6], and dengue virus type 1 (DENV-1) sequences in laboratory reared mosquitoes experimentally infected with DENV-1 [7]. Using *de novo* next generation sequencing, Makoto Kuroda *et al.* showed that the etiologic agent identified in a deceased pneumonia patient was, in fact, the pandemic influenza A H1N1 virus, rather than that originally assumed to be pneumococcus [8].

These studies highlight the power and feasibility of high throughput sequencing techniques for detection of unsuspected or novel etiologic agents. The sequencing technologies offer distinct advantages over traditional viral detection and surveillance methods that generally require prior knowledge of the etiologic agents, as well as depending on virus-specific primers, probes or antibodies. These traditional techniques are, therefore, unsuitable in situations where the causative agent of an outbreak is entirely novel, or is a pathogen variant with several mutations to key priming regions. Hence, high throughput sequencing techniques provide a powerful new opportunity for surveillance and discovery of novel pathogens. The techniques provide a cost-effective mechanism for massive parallel sequencing generating extreme sequencing depth, whilst providing multiplex analyses for etiologic agent identification.

Mosquito-borne infectious diseases have been emerging and re-emerging in many areas of the world, especially in tropical and subtropical areas where agents such as West Nile virus (WNV), dengue virus (DENV), chikungunya virus (CHIKV) and yellow fever virus (YFV) are present. Surveillance of infectious agents carried by mosquitoes is important for predicting the risk of vector-borne infectious disease outbreaks. Recently, a new strategy based on small interfering RNA (siRNA) immunity to virus infection was proposed for detecting novel RNA viruses in laboratory reared drosophilae and mosquitoes, as well as RNA/DNA viruses in plants using high throughput sequencing techniques [9], [10]. Prompted by these results (in laboratory reared insects and plants by deep sequencing and assembly of small RNAs isolated from the host organisms), we explored the feasibility of using this approach to identify viruses from wild-caught mosquitoes. Our findings show for the first time that high throughput sequencing of small RNAs can detect both RNA- and DNA viruses in wild-caught insects, thus supporting the feasibility of employing this approach for surveillance purposes.

## Results

### Standard small RNA analysis

For each mosquito species, Solexa high throughput sequencing generated about 40 million individual sequencing reads with base quality scores. After removing the sequencing adaptor and artificial junk sequences containing simple repeats of nucleotides (i.e., AAAAA…, GCGCGC…), or multiple unresolved nucleotides, which were resulted from sequencing procedures, mappable sequences were generated. By mapping to the miRNA database, we identified about 200 known miRNAs for each mosquito species. Using miRNA prediction software, one to two thousand miRNA candidates were predicted (Table 1).

**Table 1:** Statistics of standard small RNA analysis.

Samples	Raw reads	Mappable reads	Known miRNA	Predicted miRNA
*C. tritaeniorhynchus*	38,193,479	11,781,779	192	2298
*C. pipiens molestus*	50,616,662	17,723,487	201	845
*A.. sinensis*	45,936,670	8,918,988	205	2462

### Virus sequence detection

We performed BLAST analysis (using the blastn program) to identify potential viral sequences in the cleaned unique sequences. Preliminary results revealed that a large number of unique sequences in the *Culex pipiens molestus* sample shared identity with three other viruses, namely *Aedes albopictus* Parvovirus (GenBank Accession: X74945), *Anopheles gambiae densonucleosis* virus (GenBank Accession: EU233812), and *Aedes aegypti densovirus* strain 0814616 (GenBank Accession: FJ360744). Further analysis demonstrated that the matched *A. albopictus* Parvovirus sequences were also present in the *A. gambiae densonucleosis* virus genome and the *A. aegypti densovirus* strain 0814616 genome. The *A. gambiae densonucleosis* virus and *A. aegypti densovirus* strain 0814616 shared most of their matched sequences. Sequence alignment showed that these three viruses exhibited more than 80 percent sequence identity, indicating that a virus with homologous sequences to these three viruses was present within the *C. pipiens molestus* sample. For the other two samples (*C. tritaeniorhynchus* and *A. sinensis*), no significant amount of sequence was found that corresponded to any specific virus. To discover potential novel viruses which may be remotely related to known viruses, a BLAST strategy proposed in the literature [9] was adopted. This strategy employs the tblastx search to ensure identification of viruses based on amino acid sequences. However, this analysis did not reveal any additional viral sequences in any of the three mosquito samples tested.

### Small RNA sequence analysis of the newly identified virus

**Figure 1:**
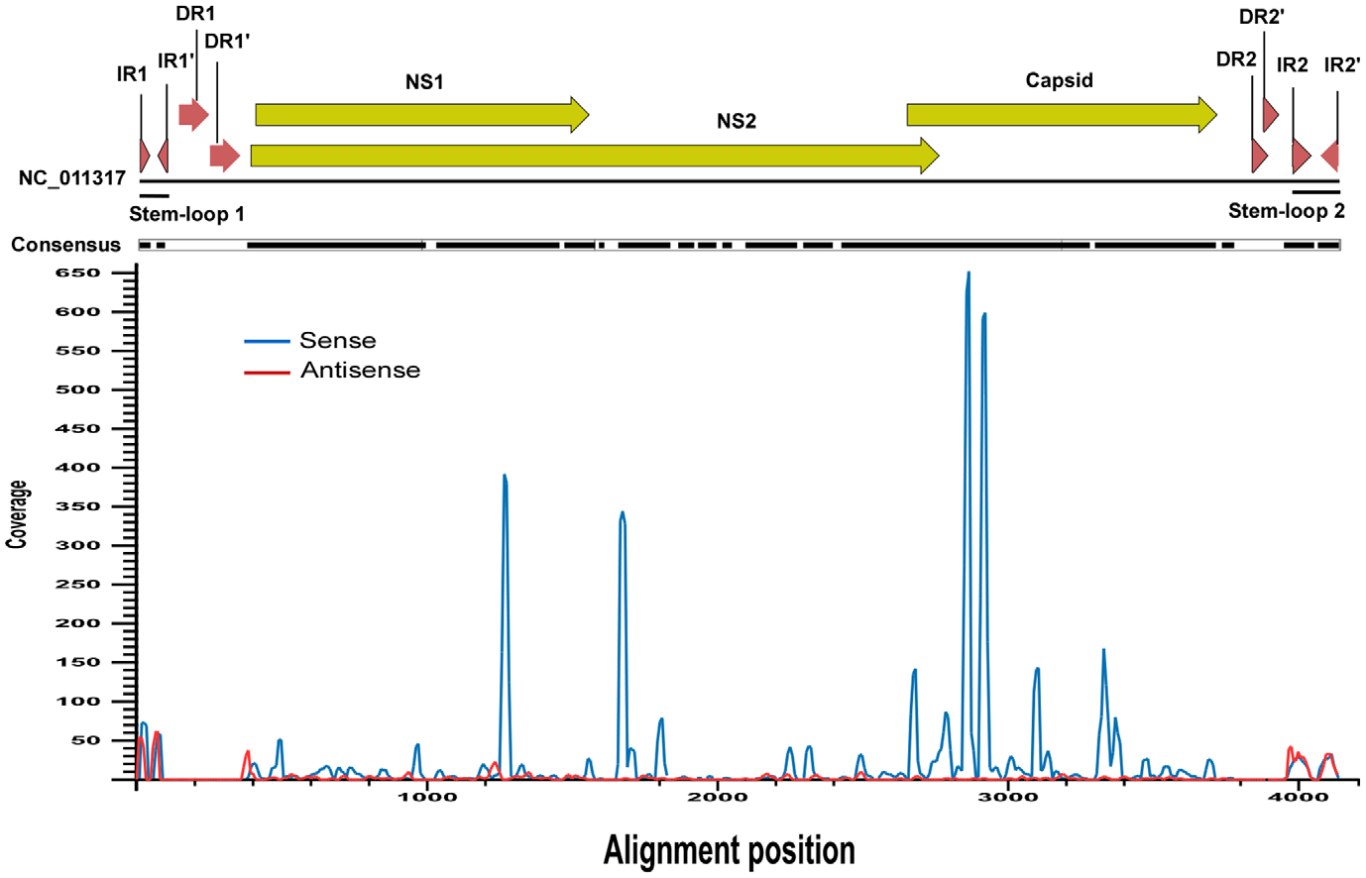
Mapping of sequencing reads onto the densovirus genome. Cleaned sequence reads were mapped onto the densovirus genome (GenBank accession number NC_011317). The 4139 bp genome contains three open reading frames (NS1, NS2 and Capsid gene, represented by long bold yellow arrows) which are flanked by inverted repeats (IR, represented by short red bold arrows) and direct repeats (DR, represented by short bold red arrows) at both termini of the genome. The gapped lines represent the regions where sequenced reads were mapped. The blue peaks in the lower part indicate the coverage (occurrence frequency) of the sense strand reads and the red peaks indicate the coverage of the antisense strand reads.

**Figure 2:**
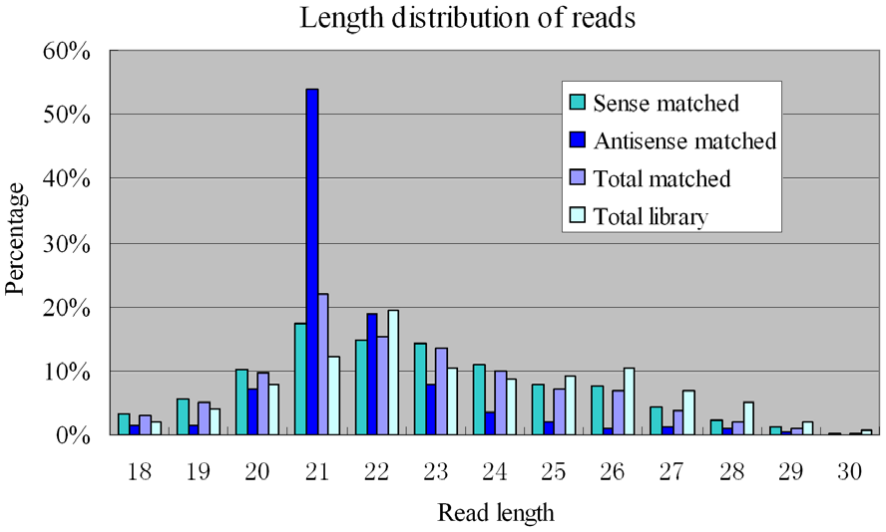
Length distributions of small viral RNAs. A plot of the percentage of the different lengths of the small viral RNA's obtained. The most frequent length of the small viral RNAs was 21 nt.

To characterize small RNA sequences with homology to the viral genomic sequences, mappable sequence reads were assembled using three viral genomes as references (i.e., *C. pipiens molestus*, *C. tritaeniorhynchus* and *A. sinensis*) with CLC Bio (Katrinebjerg, Denmark) using the default parameters. The results showed that the small RNA reads overlapped which allowed contig assembly. Of the three viruses, *A. gambiae densonucleosis* had the most mapped reads (4481) and the longest assembled consensus sequence (3248 bp) which covered 78.5% of the whole genome (4139 bp) (Figure 1 and file S1). The overall similarity between the newly identified virus and the *A. gambiae densonucleosis* virus was about 98% (3182/3248). The distribution of the lengths of the matched small viral RNAs showed that the majority (>60%) of them were 20–24 nt in length, with a peak distribution of 21 nt, while the total library small RNA (majority of them were endogenous siRNA) displayed a peak distribution of 22 nt (Figure 2). This is consistent with the discovery in sindbis infected mosquitoes [11] and virus infected Drosophila OSS cells [9], where the matched viral siRNAs had a peak distribution at 21 nt, while the endogenous siRNA in Drosophila [12], [13] and the total library small RNA in mosquitoes [11] had a peak length of 21∼22 nt. Most of the small RNAs distributed along three viral transcripts (e.g. NS1, NS2 and Capsid), with more than 80% of the transcript length covered. The viral small RNA +/− strand ratio approximated 7∶1 (3933/548), indicating that these molecules were largely derived from the viral RNA transcripts. The mechanism for biogenesis of the – strand small RNAs in mosquitoes is currently unknown.

### Characterization of high frequency small viral RNA sequences

Although most of the viral coding transcripts (NS1, NS2, and Capsid) were covered by the small viral RNA sequences, the small RNAs were not evenly distributed along the transcripts. There were 10–20 sites with relatively high coverage and 4 of these had very high coverage indeed (greater than 320× coverage compared with the average coverage of 24×) (Figure 1). The core sequences of the high frequency reads were 20–22 nt in length, with 3 or 4 adenosine bases located at the 5′ terminus (Table 2). The two most frequently occurring sequences (with greater than 600× coverage) were in the coding region of the viral capsid protein gene, while the other two medium high copy number sequences were located in the NS1 and NS2 genes, respectively. The biological relevance as well as the biogenesis of these high frequency reads requires further investigation.

**Table 2:** Characteristics of high coverage sequences mapped on the genome.

Core sequence	Core coverage	Core region	Extended region	Genome location
AAAGAGGACTGGAGATACAT	389	1260–1279 (20 bp)	1251–1286 (36 bp)	NS1
AAAAGATGCGGACAACGTAAAC	344	1662–1683 (22 bp)	1653–1689 (37 bp)	NS2
AAAATACTTGGACTTCAATT	652	2851–2870 (20 bp)	2844–2882 (39 bp)	Capsid
AAACGGCAGGATTCTGGGCA	594	2907–2926 (20 bp)	2900–2938 (39 bp)	Capsid

### Identification of small RNA sequences in the direct repeat region within 5′ and 3′ UTRs

The densovirus genome contains two-pairs of inverted repeats, which constitute two stem-loop structures at the 5′ and 3′ untranslated regions of the genome termini (Figure 1). It also contains two pairs of direct repeats in close proximity to those inverted repeats. All of these repeats are located in the untranscribed regions at the genome termini. It is interesting to note that no small RNAs were mapped to the untranscribed regions, although large numbers of reads mapped to the four inverted sequences (with a coverage greater than 50×), but not the direct repeats (Figure 1, Table 3). Sequencing was performed on small RNA fragments, therefore, the fact that no reads mapped to the untranscribed region was not unexpected. The fact that reads mapped to the untranscribed 5′ and 3′ inverted repeat regions indicates that those inverted repeat regions may be transcribed by an unknown mechanism. Since the terminal stem-loop structures are usually involved in viral genome replication, it is possible that transcripts from the stem-loop regions are involved in virus replication (e.g. as primers for genomic DNA synthesis).

**Table 3:** Characteristics of sequences mapped to non-coding regions.

Core sequence	Core coverage	Core region	Extended region	Genome location
TGATACGGATACTGTAAGATA	132	13–33 (21 bp)	11–38 (28 bp)	IR1
TGTATCTTACAGTATCCGTAT	117	64–84 (21 bp)	60–88 (29 bp)	IR1'
GATCCCCGTGTGAGCCGATAGGCGAGGATCGAA AGCCCAAATTTTGCTGACGTCACCTCACACACATA	65	3964–4031 (68 bp)	3950–4053 (104 bp)	IR2
AAAGCTTTTGGTATGTGTGTGAGGTG ACGTCAGCAAAATTTGGGCTTTCGATC	68	4075–4125 (51 bp)	4075–4125 (73 bp)	IR2'

It is notable that the high coverage small RNAs in the coding regions and the small RNAs mapping to the non-coding stem-loop regions are both highly conserved (0/385 nucleotide difference), compared to the genome as a whole which is roughly 2% different to the reference densovirus (EU233812). Such high evolutionary conservation suggests that these sequences are of functional importance.

### Validation of viral infection by polymerase chain reaction

**Figure 3:**
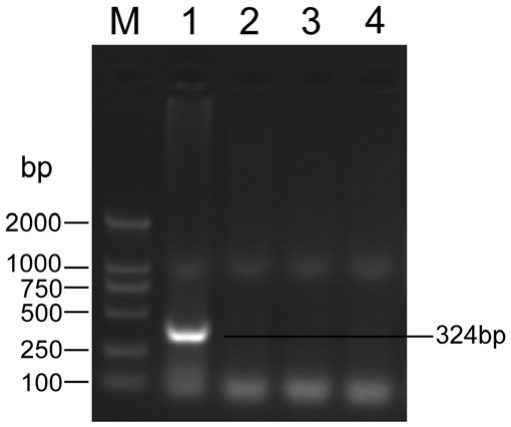
PCR amplification of densovirus sequence from the mosquito DNA. Detection of densovirus in wild-caught mosquitoes with PCR using primers designed from the virus sequences assembled with the small RNAs. M, DNA molecular weight markers. 1, *Culex pipiens molestus*. 2, *Culex tritaeniorhynchus*. 3, *Anopheles sinensis*. 4, distilled water negative control.

To validate the presence of a viral infection, a standard PCR was conducted using total DNA extracted from samples of the three mosquito species. Gel electrophoresis demonstrated that a DNA band of the appropriate size had been amplified in the *C. pipiens molestus* mosquito sample, but not from *C. tritaeniorhynchus* or *A. sinensis* (Figure 3). Sequence analysis of the PCR product revealed same sequence as that assembled by the small RNAs. These results, therefore, confirm the existence of a densovirus in *C. pipiens molestus*, but not in *C. tritaeniorhynchus* and *A. sinensis*. We have called this densovirus *Culex tritaeniorhynchus densovirus* YN2009.

### Phylogenetic analysis of the newly identified densovirus

**Figure 4:**
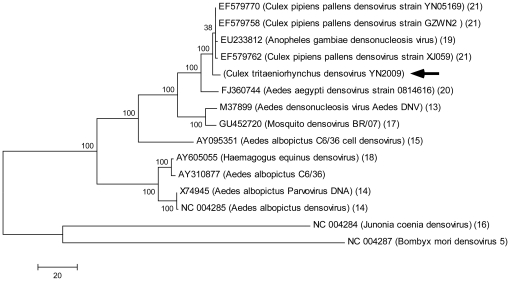
Phylogenetic analysis of the isolated densovirus. The phylogenetic tree was generated using Mega 4.0 with maximum parsimony and bootstrap 500. Reference densovirus strains were selected after blasting the NCBI NT database with a 398 bp fragment assembled with the small RNAs. Numbers in parentheses indicates the reference number of the particular virus stain [14], [15], [16], [17], [18], [19], [20], [21], [22]. The virus strain identified in this work has been assigned the name *Culex tritaeniorhynchus densovirus* YN2009 (indicated by a solid arrow). Scale bar represents the number of nucleotide substitutions.

To understand the evolutionary status of the densovirus identified here, a phylogenetic tree was generated with Mega 4.0 using maximum parsimony and bootstrap 500 methods (Figure 4). The reference densovirus strains [14], [15], [16], [17], [18], [19], [20], [21], [22] were downloaded from GenBank after blasting the NT database with a 398 bp segment assembled from the small RNAs. The phylogenetic tree obtained infers that newly identified *Culex tritaeniorhynchus densovirus YN2009* is a close relative of the mosquito densoviruses prevalent in South and Southwest China.

## Discussion

High throughput sequencing as a next generation sequencing technology has been developing rapidly during the last few years and has found various applications in different biological and medical research fields. Recent advances in this technology have made its application easier, cheaper, more convenient and more efficient allowing it to evolve into a powerful tool for identification of novel human pathogens [3], [4], [5], [6], [7]. High throughput sequencing of small RNA's (esp. miRNA) has become routine practice, with reliable protocols and readily available reagents. Due to the short length of the small RNA molecules, sequencing is even faster and cheaper than standard high throughput sequencing using longer DNA or RNA fragments. This makes high throughput sequencing of small RNA an attractive method for pathogen detection in plants and insects based on siRNA, an innate defense mechanism of plants and insects [9], [10]. Detection of viruses in laboratory reared insects [9] or experimentally infected mosquitoes have been reported [7]. Our work shows that high throughput sequencing is suitable for detecting viral agents in wild-caught insects.

Since siRNA defense mechanisms are triggered by the double-stranded RNA (dsRNA) sequence (and the siRNA mature forms generated from dsRNA), it is reasonable to expect that only RNA viruses which contain dsRNA as genomic RNA or replicate via a dsRNA intermediate can be identified using this strategy. This perception is consistent with previous reports where only RNA viruses were identified using small RNA sequencing techniques [7], [9]. However, our work clearly demonstrates that small RNA sequencing can also detect DNA viruses in insects, although the underlying mechanism of the biogenesis of these small RNAs is unclear. Similar findings have been reported in plants [10], but again the mechanism has not been defined. It is possible that plants and insects generate small RNAs from infected DNA viruses differently. Possible mechanisms for small RNA biogenesis from DNA viruses include, for example, the local dsRNA formed in the stem-loop structure of the viral transcripts or overlapping convergent viral transcripts [23], [24]. In the case of densoviruses, there seem to be no overlapping convergent transcripts [25], [26] and no obvious stem-loop structure has been identified in densovirus transcripts.

An alternative explanation for the small RNAs derived from the DNA virus may be degradation of virus transcripts. However, this hypothesis cannot explain at least two things: one is the very high incidence of some small RNAs that have 3–4 adenines at the 5′ terminus, the other is the biogenesis of the small RNAs that map to the inverted regions of the genomic termini. These are predicted to form a T- or Y-shaped structure that may participate in genome packaging signaling or replication initiation [26]. It is interesting to note that a longer length direct repeat was located very close to each inverted repeat (Figure 1), but no small RNA mapped onto the direct repeats themselves. The function of the inverted and direct repeats, and how the small RNAs are generated from the inverted repeats but not from the direct repeats, remain interesting questions to be answered. To this end, we provide all the original data containing the read sequences of the virus small RNAs as a supplementary file to this paper (file S1).

Mosquitoes are the most important vector of WNV, DENV, CHIKV and YFV, and controlling mosquito populations is an important way of preventing epidemics of these life-threatening diseases. Among the many approaches to mosquito control [27], environmentally friendly densoviruses have been considered as a biological control agents [20], [26], [27], [28]. Field trials using a densovirus that infects *A. aegypti* mosquitoes showed that the virus had significant efficiency, although most densoviruses take 2–20 days to kill their insect hosts [29], making this agent unsuitable for commercial use. However, with the advent of genetic engineering, it might be possible to generate genetically modified densoviruses that could be effective mosquito control agents. Better understanding of the biology of densoviruses and their relationship with mosquito host immunity could therefore be of practical importance for addressing disease control.

Traditional generic methods for identifying and characterizing novel viral diseases have included electron microscopy, virus isolation in cell culture, immunological approaches and PCR. Recently technologies such as diagnostic microarrays and mass spectrometry have been proposed as generic tools for identifying viruses [30], but all these methods require some prior knowledge of the agents to be identified. With the advent of next generation high throughput parallel sequencing platforms, the possibility of random metagenomic sequencing of diseased samples with the object of identifying new putative pathogens has emerged [6], [31]. However, elimination of host nucleic acid is critical to boost any pathogen signal toward the detection threshold. In addition, the danger of missing extremely low titer viruses is still a possibility with these systems. By comparison, small RNA sequencing requires neither viral particle purification nor viral nucleic acid sequence amplification. With the advantages of high throughput, high speed, low cost and greatly simplified methodologies, small RNA sequencing can now be used more widely to identify known viruses as well for novel virus discovery.

Although the densovirus identified here was not a significant etiologic agent, this discovery proves that the approach is applicable not only for discovery of RNA viruses, but also DNA viruses in mosquitoes. Currently all known human pathogenic viruses found in mosquitoes are RNA viruses, but this does not preclude DNA viruses from using mosquitoes as vectors for human, animal or plant diseases. Indeed the African swine fever virus is an arthropod-borne double-stranded DNA virus [32] which causes a lethal hemorrhagic disease in domestic pigs.

In conclusion, our study is the first to explore the application of convenient small RNA high throughput sequencing for virus discovery in wild-caught vectors. Our results suggest that small RNA sequencing is able to identify not only RNA viruses, but also DNA viruses in wild-caught mosquitoes, obviating the need for culture-based virus isolation or for prior knowledge of the etiologic agent. These results suggest that small RNA high throughput sequencing could be an ideal tool for surveillance of novel emerging viral disease or even non-viral infectious diseases.

## Materials and Methods

### Mosquito collection

The mosquitoes, including *Culex tritaeniorhynchus*, *Culex pipiens molestus*, and *Anopheles sinensis* were collected from Yunnan province, China, in 2009. The samples were stored in liquid nitrogen until RNA extraction. No specific permits were required for the described field studies; the samples collected were not privately owned or protected and did not involve endangered or protected species.

### Small RNA library preparation and sequencing

Prior to RNA extraction, mosquitoes were cleaned in sterilized water and dried with hygroscopic filter paper. Mosquitoes of the same species were pooled together. Total RNA was extracted separately from the different mosquito species using the Total RNA Purification Kit (LC Sciences, Houston, USA), according to the manufacturer's instructions. The quality of total RNA was analyzed on an Agilent 2100 Bioanalyzer system and by denaturing polyacrylamide gel electrophoresis. A small RNA library was generated according to the Illumina sample preparation instructions [33]. Briefly, total RNA samples were size-fractionated on a 15% tris-borate-EDTA-urea polyacrylamide gel. RNA fragments 15–50 nt long were isolated, quantified, and ethanol precipitated. The SRA 5′ adapter (Illumina) was ligated to the RNA fragments with T4 RNA ligase (Promega). The ligated RNAs were size-fractionated on a 15% tris-borate-EDTA-urea polyacrylamide gel and 41–76 nt long RNA fragments were isolated. Next the SRA 3′ adapter (Illumina) ligation was performed, followed by a second size-fractionation using the same gel conditions as described above. The 64–99 nt long RNA fragments were isolated by gel elution and ethanol precipitation. The ligated RNA fragments were reverse transcribed to single-stranded cDNAs using M-MuLV (Invitrogen) with RT-primers (as recommended by Illumina). The cDNAs were amplified with pfx DNA polymerase (Invitrogen) using 20 PCR cycles and the Illumina small RNA primer set. PCR products were purified on a 12% tris-borate-EDTA polyacrylamide gel and a slice of gel containing cDNAs of 80–115 bp was excised. This fraction was eluted and the recovered cDNAs were precipitated and quantified on the Nanodrop (Thermo Scientific) and on the TBS-380 mini-fluorometer (Turner Biosystems) using PicoGreen® dsDNA quantization reagent (Invitrogen). The concentration of the sample was adjusted to 10 nM and 10 µL used for the sequencing reaction. The purified cDNA library was used for cluster generation (on the Illumina Cluster Station), and then sequenced on the Illumina GAIIx machine, following the manufacturer's instructions. Raw sequencing reads were obtained using the Illumina Pipeline v1.5 software following sequencing image analysis by the Pipeline Firecrest Module and base-calling by the Pipeline Bustard Module.

### Standard small RNA analysis

Clean-up of the raw data and subsequent small RNA mapping and prediction were performed with a proprietary software package, ACGT101-miR v3.5 (LC Sciences, Houston, Texas). First, low-quality reads were removed from the raw reads. After removal of the adaptor sequences, and filtering of the low quality reads and simple artificial sequences, the mappable reads were extracted and the unique sequences generated by collapsing the identical sequences, with the occurrence count of each unique sequence as the unique sequence tag. These unique sequences were compared with the sequences of non-coding RNAs (rRNA, tRNA, snRNA, snoRNA) available in Rfam (http://www.sanger.ac.uk/software/Rfam) and in the GenBank non-coding RNA database (http://www.ncbi.nlm.nih.gov/) to clarify degradation fragments of non-coding RNA. In addition, all sequences were mapped to miRNA sequences from the miRNA database, miRBase 16.0 (http://www.mirbase.org/).

### Viral sequence detection using the BLAST program

BLAST searches were conducted to identify the virus sequences in the cleaned unique reads using the blast-2.2.22 package [34]. Due to the large amount of high throughput sequencing data, we formatted the sequencing reads, (using the command formatdb that is included in the BLAST package), as a BLAST database and used the viral sequences downloaded from the EMBL website (http://www.ebi.ac.uk/embl/) as a query, in order to expedite the BLAST process. BLAST results were then analyzed manually to screen for potential virus sequences.

### PCR confirmation of viral infection

Total mosquito DNAs were extracted with TRIzol reagent (Introgen, Carlsbad, CA) according to the manufacturer's instructions. A pair of primers (forward primer: 5′-ATA AAT TGA TCA GTC GTC CTC CAA C-3′; reverse primer: 5′-CTT GGG ATC ATT TCG GTC ATA T-3′) were selected from the viral sequence assembled with the mappable reads. The PCR was conducted in a 50 µl reaction mixture containing 1×Easy Taq PCR SuperMix (TransGen Biotech, Beijing, China), 1 µM each of the forward and reverse primers and 10 ng of template DNA. After pre-denaturation at 94°C for 3 minutes, 35 cycles of amplification (30 sec denaturation at 94°C, 30 sec annealing at 55°C, and 60 sec polymerization at 72°C) were performed, followed by a final incubation at 72°C for 5 min. PCR products were visualized on a 1% agarose gel stained with ethidium bromide.

## Supporting Information

File S1 **Reference assembly of the small RNA with the densovirus genome (GenBank accession number NC_011317) as the reference sequence.** The read alignment is saved in the ace file format and can be viewed with common read alignment program like tablet (freely available on http://bioinf.scri.ac.uk/tablet/). All the read sequences can be retrieved from the ace file with any text editing program.
(ACE)
